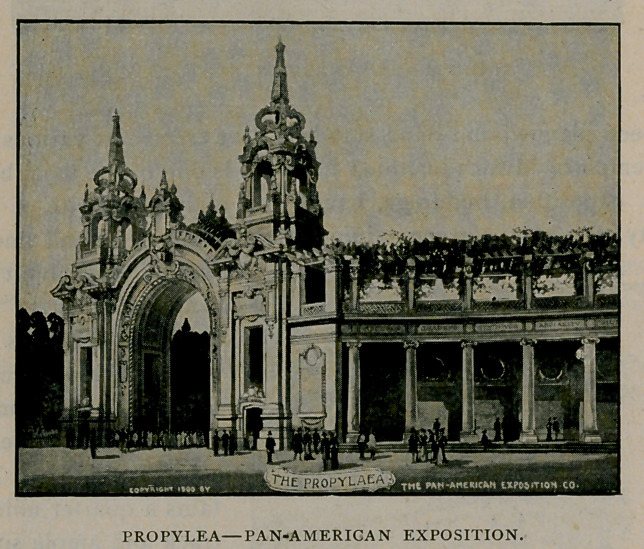# The Pan-American Exposition of 1901

**Published:** 1901-01

**Authors:** 


### Special Article.

### THE PAN-AMERICAN EXPOSITION OF 1901.

A sketch, compiled and arranged by the Editor.

CAPTAIN JOHN M. BRINKER, of Buffalo, a leading citizen, capitalist, and business man, may be said to be the father of the Pan-American Exposition, to be held at Buffalo from May 1 to November 1, 1901. This will be the first attempt to present within a single enclosure the enterprise, education, art and general business progress of the peoples of the Western Hemisphere. When Captain Brinker returned from the Nashville exposition, four or five years ago, filled with the idea of establishing an exposition representing all America, he could scarcely have dreamed that his enterprise would have reached the wonderful proportions it has already attained, to say nothing of the magnificence of its ultimate prospective magnitude.

WILLIAM I. BUCHANAN,

DIRECTOR-GENERAL,

PAN-AMERICAN EXPOSITION.

During the summer of 1897, while President McKinley was attending the meeting of the Grand Army of the Republic, he drove the stake that located the exposition grounds on Niagara River, near La Salle. Some two years ago, however, when the exposition had assumed definite shape, this location was thought untenable and one nearer to Buffalo, on the north side of the city, near the park, was chosen. This has been improved, laid out, built upon and beautified, until now, five months before the opening of the exposition, the buildings and grounds present a marvelously attractive appearance in architecture and landscape, in color and plan, in beauty and solidity, that challenges the admiration of a daily throng of visitors from all parts of the world.

Having obtained subscriptions, appropriations, loans and contributions; then having elected officers and selected grounds, the next most important question thrust upon the managers was the election of a director-general. Viewed from every standpoint it appeared to the board that the Honorable William I. Buchanan was in every way qualified for the post. Mr. Buchanan is a linguist of fluency, grace and finish; having served as minister to the Argentine Republic, he is familiar with the South American Continent, with its people and its customs and with the products of the several countries. Moreover, Mr. Buchanan is an organiser of great capacity, an executive officer of force and urbanity, and is every way fitted by experience and training to serve in the capacity of director-general of the first Pan-American exposition. The management is to be congratulated upon securing his services as the executive head of the exposition.

OFFICIAL EMBLEM.

PAN-AMERICAN EXPOSITION.

The choice of an official emblem presented a problem of no small import. Competition was invited and the design, which we illustrate, was finally chosen.

It was drawn by Raphael Beck of Buffalo, and it was accepted as the most artistic and suitable from several hundred designs submitted. It has the special merit of effectively symbolising one of the chief purposes of the exposition, which is to bring into closer social and trade relationship the republics, states and territories of North and South America. The emblem shows a fair maiden typifying the North, extending a kindly hand to clasp that of her brunette sister of the South, thus forming a bond of continental sisterhood and establishing a unity of sentiment and interest among the countries of the Western Hemisphere.

The next important step was the construction of the buildings; designs must be drawn, contracts let and many preliminary details arranged before a start could be made. Notwithstanding all this the work was commenced in the spring of 1899, and has steadily progressed in spite of strikes and storms, until a group of buildings grand in architecture, heroic in proportion, and beautiful in color effect have resulted.

The Service Building was the first structure erected on the grounds. It is the administrative headquarters of the exposition, all the officers whose presence is required upon the grounds having their headquarters there. All around the Service Building the grounds have been given the horticulture and floral decoration that

SERVICE BUILDING—PAN-AMERICAN EXPOSITION.

will embellish the entire exposition plot. This decorative work gives the visitor a foretaste of the wonderful beauty that is to characterise the exposition.

The United States and several of the leading commonwealths are erecting buildings in which will be exhibited the products of the several states and dependencies.

The United States Government is spending $500,000 upon its group of three great buildings and the exhibits to be contained in

UNITED STATES GOVERNMENT BUILDING—PAN-AMERICAN EXPOSITION.

them. The several departments of the government will make very complete displays, and in addition to these will be new exhibits from the Hawaiian and Philippine Islands, Tutulia, Guam, Porto Rico and Cuba. Among the more important features will be the great exhibit of fishes, the Weather Bureau, exhibits from the mint, naval and war exhibits and many others. Of particular interest will be the big gun exhibit,—a group of three immense pieces of ordnance being mounted immediately at the north of the main building.

NEW YORK STATE BUILDING—PAN-AMERICAN EXPOSITION.

The New York State Building is to be a permanent edifice. The material used in its construction is white marble, and the style of architecture is that of a Doric Temple. At the close of the exposition this magnificent structure will become the property of the Buffalo Historical Society. It will cost upwards of $170,000—the state contributing $100,000 of the $300,000 appropriated by the legislature,

MACHINERY BUILDING—PAN-AMERICAN EXPOSITION.

and the Historical Society and the City of Buffalo contributing the remainder.

Machinery and transportation always possess great interest to every intelligent person.

The Machinery Building covers an area of about four acres, and will contain a very wonderful display of modern machinery of American invention, showing the progress that has been made within the last few years. The large amount of automatic and special machinery used in American factories and mills, will form a most interesting study to all who are interested in the products of the Western world. The transportation exhibits will include all of the very latest specimens of locomotives, cars and railroad appliances. These will be sheltered in a special building in connection with the large railway station at the northern end of the exposition grounds. The ordnance exhibits will be made in connection with the machinery exhibits, and will show very remarkable progress in the manufacture of ordnance in the western world. This department is distinct from the war and naval exhibits of the Federal Government, and will be sheltered in a special building.

A TROPICAL COURT—PAN-AMERICAN EXPOSITION.

This illustration shows the court designed for the Machinery and Transportation Building. It is one of the many beautiful features of the exposition, consisting of an open space, made brilliant with flowers, and a sparkling fountain in the center of a cool and clear basin of water. About this court will be arranged comfortable seats where the visitor may rest and enjoy the beauty of the shrubs and flowers.

MANUFACTURES AND LIBERAL ARTS BUILDING—PAN-AMERICAN EXPOSITION.

The Manufactures and Liberal Arts Building covers more than four acres, and will contain the very latest productions of the mills and factories of the United States and other countries of the Western

Hemisphere. Exhibits showing the processes of manufactures will constitute a very interesting feature of this division.

ELECTRICITY BUILDING—PAN-AMERICAN EXPOSITION.

Electricity and everything connected with it will naturally possess absorbing interest to almost every visitor. The fact that so much power brought from Niagara Falls is used in Buffalo is in and of itself a marvel. Street cars, printing presses and other machinery is driven by it, while the power used upon the exposition grounds will be generated at Niagara Falls, twenty-two miles away.

ELECTRIC TOWER—PAN-AMERICAN EXPOSITION.

At the Pan-American Exposition will be shown the largest display of electrical machinery and appliances ever presented. Nearly every article will be of the very latest design, and the visitor may expect novelties without number in this interesting division. The Electricity Building is of very rich and beautiful design, having a broad loggia on the southern side while the roof line is broken with domed towers.

The Electric Tower, 375 feet high, will be the center piece of the exposition. The beauty of this tower is beyond description. The entire exterior is of richly molded work and many artistic groups of sculpture will adorn it at salient points. It will stand in a broad basin and from a niche in its southern face will gush a cascade 30 feet wide and 70 feet high.

ALBRIGHT GALLERY OF ART—PAN-AMERICAN EXPOSITION.

The illumination of this tower at night will be particularly beautiful and wonderful.

The Albright Art Gallery will cost more than $400,000. It is to be a permanent building of white marble. After serving the purposes of an art gallery for the exposition it will become the permanent

TEMPLE OF MUSIC—PAN-AMERICAN EXPOSITION.

home of the public art treasures of Buffalo. It is the gift of Mr. J. J. Albright, a citizen of Buffalo.

Music is to be one of the great features of the exposition. Contracts have been made for a series of concerts by Sousa's Band, and the Mexican Government Mounted Band of sixty-two men. Many other famous organisations will be engaged. Large music gardens

STADIUM—PAN-AMERICAN EXPOSITION.

have been planned and band stands will be erected at various points. The Temple of Music illustrated herewith is one of the most beautiful of the exposition buildings, having an auditorium with a seating capacity of 2,200, and containing one of the largest and finest pipe organs ever built in the United States.

CORNER OF STADIUM.

PAN-AMERICAN EXPOSITION.

Athletic sports will be well brought out and cared for in the Stadium where there will be seats for 12,000 people. It contains a quarter mile racing track and ample space for all the popular athletic games. Here also will be the displays of live-stock, automobiles and other road vehicles, farm and road machinery in motion. The large space beneath the seats will be used for exhibits.

This picture of a corner of the Stadium shows the massive and beautiful character of the architecture. This will be a very large structure and during the exposition season there will be held an athletic carnival of particular interest. The entrance to the Stadium is a large building having an arcaded arrangement on the ground floor. The upper floors are to be used for restaurant purposes.

The illustration herewith shows the western end of the propylea. This is an architectural ornament of very beautiful and imposing design. It marks the northern boundary of the Plaza, and is designed as a screen, separating the exposition from the noise and smoke incident to the traffic of steam railways which pass the exposition grounds upon the northern side. The Propylea is 510 feet long with a massive towered entrance at each end.

PROPYLEA—PAN-AMERICAN EXPOSITION.

Within the exposition grounds are 133 acres of Delaware Park, including the Park Lake. This lake is a very beautiful body of water, and upon its shores the United States Government will erect a life-saving station, where a crew of ten men will give daily exhibitions during the exposition season, showing the uses of life-saving apparatus.

In this very imperfect outline we have given our readers but a glimpse of some of the interesting features of this wonderful exhibition. A hospital building is in course of construction, and a staff of assistants and nurses will be appointed to serve during the life of the exposition. There remain other great and beautiful buildings, and the always attractive midway, which we hope to say something of in a future issue.

## Figures and Tables

**Figure f1:**
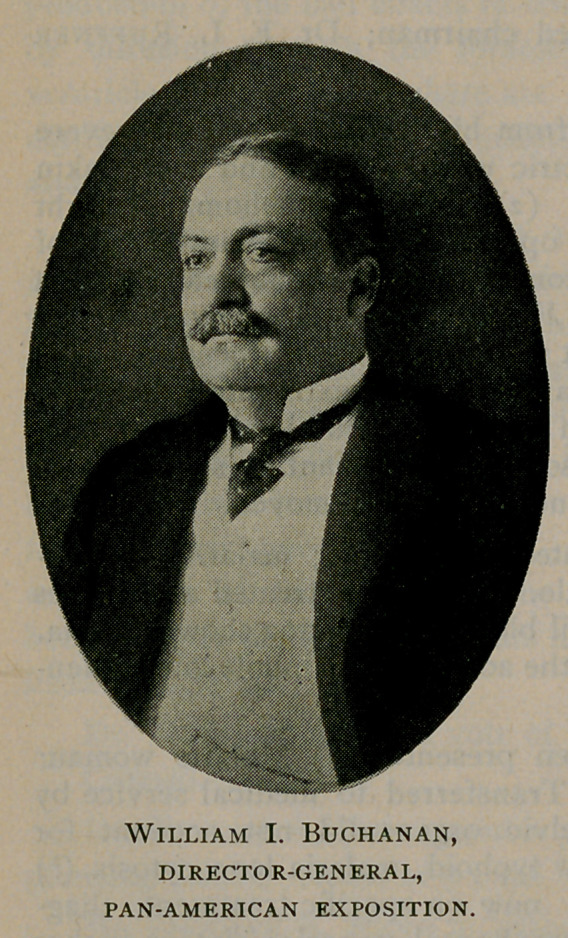


**Figure f2:**
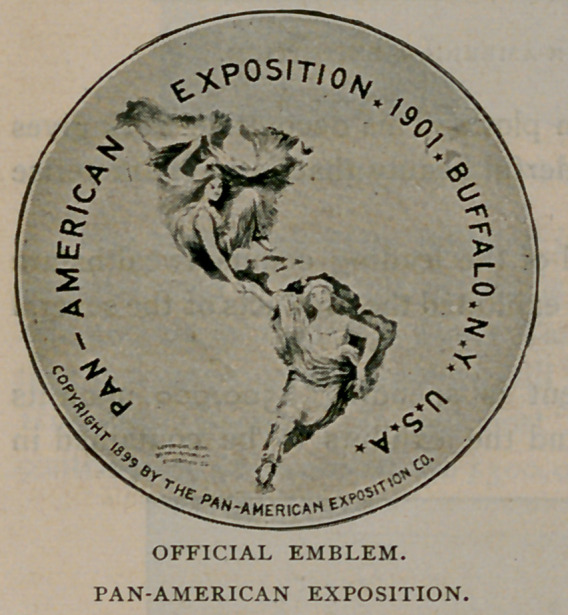


**Figure f3:**
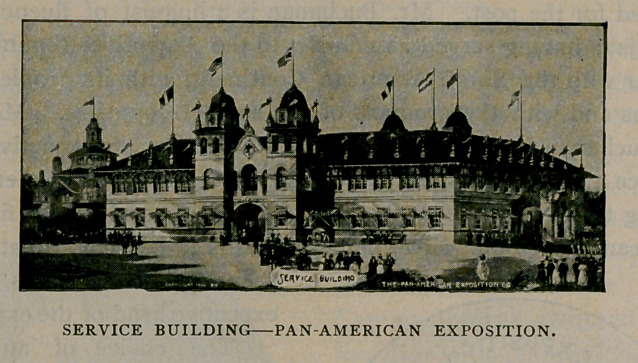


**Figure f4:**
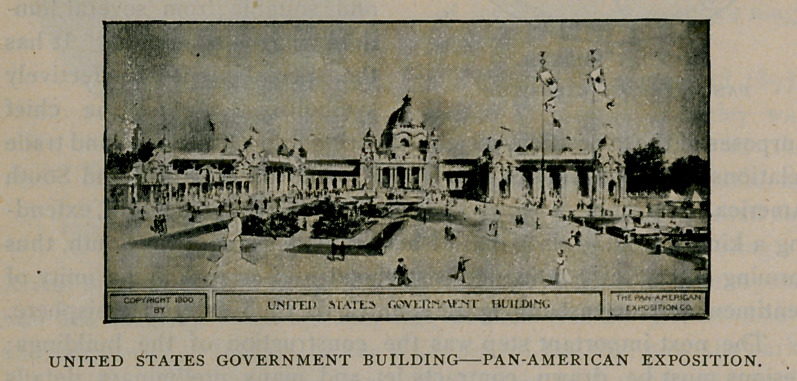


**Figure f5:**
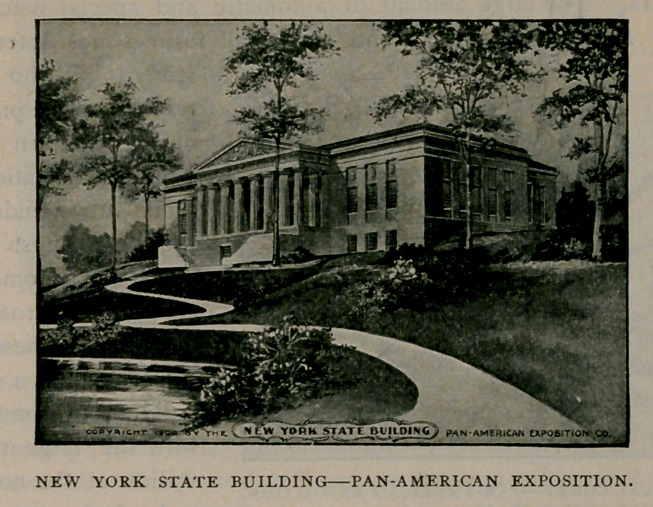


**Figure f6:**
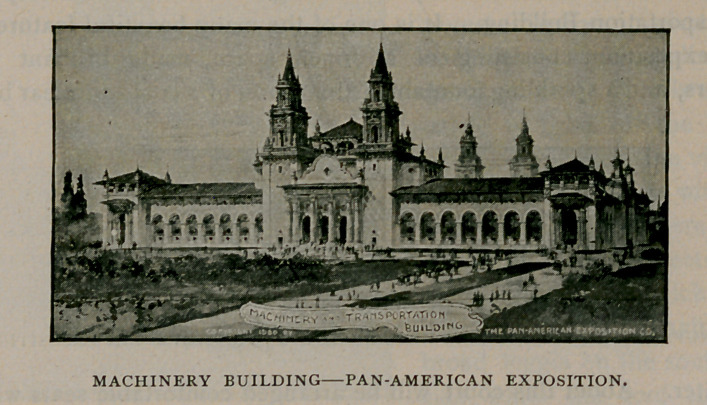


**Figure f7:**
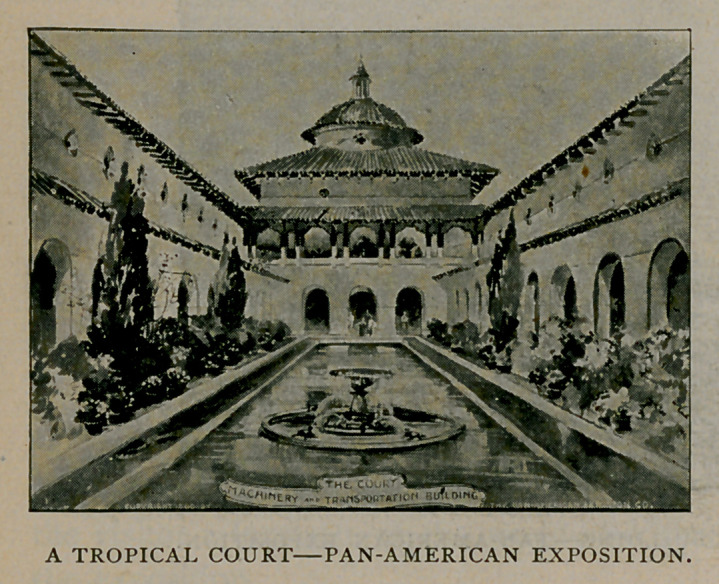


**Figure f8:**
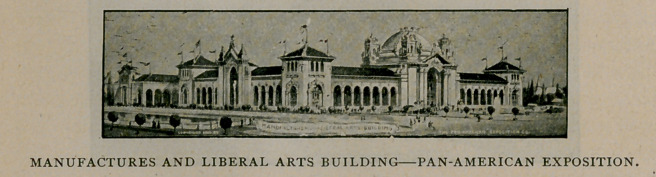


**Figure f9:**
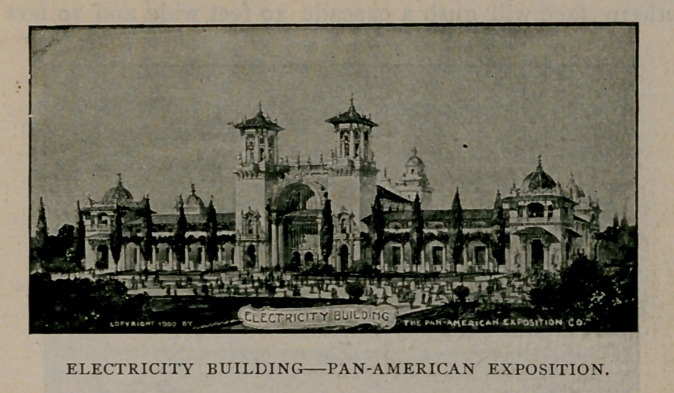


**Figure f10:**
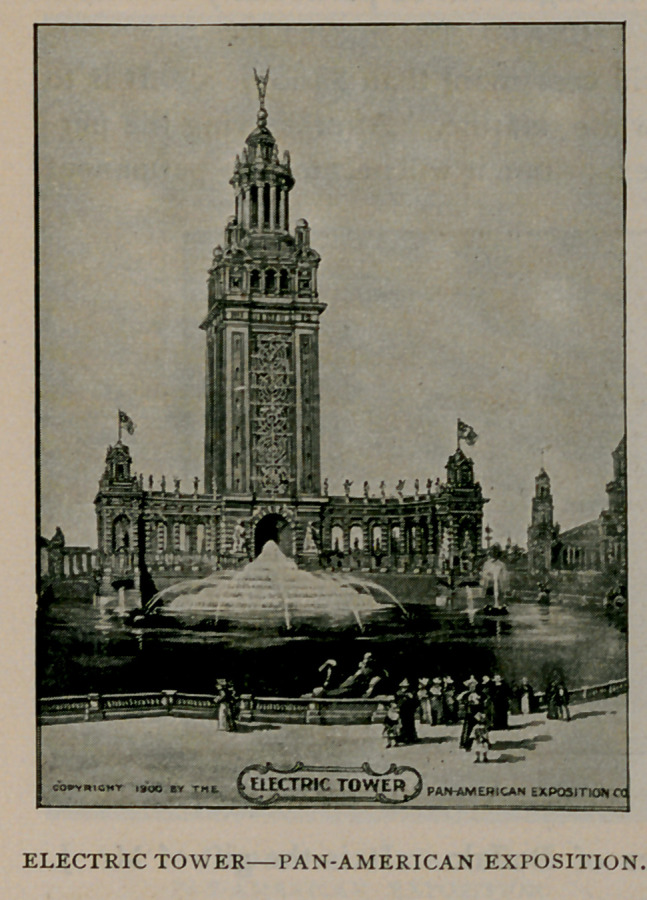


**Figure f11:**
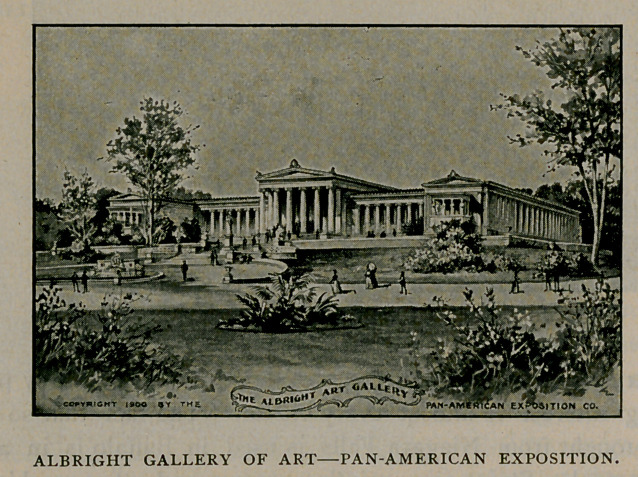


**Figure f12:**
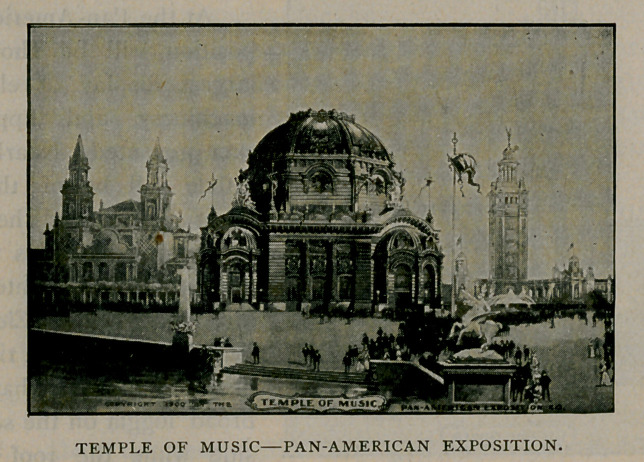


**Figure f13:**
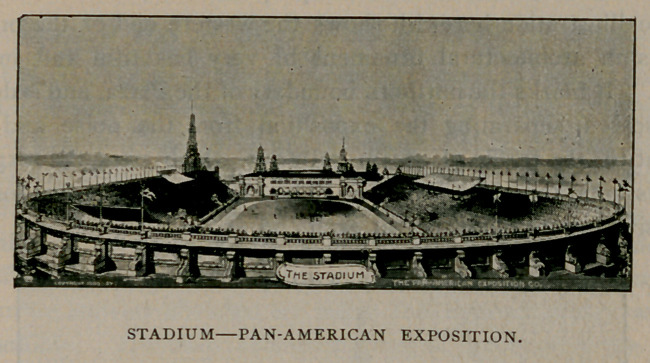


**Figure f14:**
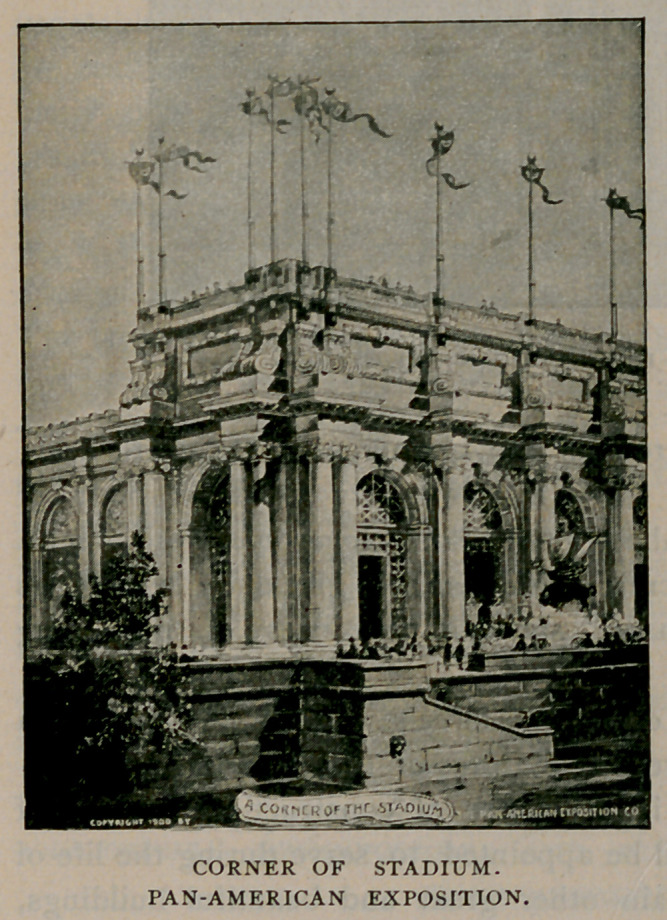


**Figure f15:**